# Diallyl trisulfide improves spinal cord ischemia–reperfusion injury damage by activating AMPK to stabilize mitochondrial function

**DOI:** 10.1186/s13018-023-04176-8

**Published:** 2023-11-06

**Authors:** Yang Sun, Dengyue Xu, Weidong Yang, Hongquan Zhang, Yi Su, Bin Gao, Xiaowei Zou, Yiming Zhong, Huanwei Sun, Liangbi Xiang

**Affiliations:** 1 Department of Hand and Foot Surgery, Central Hospital of Dalian University of Technology, No. 826, Southwest Road, Shahekou District, Dalian, 116000 Liaoning Province People’s Republic of China; 2 Postgraduate College, China Medical University, No. 77, Puhe Road, New Shenbei District, Shenyang, 110122 Liaoning Province People’s Republic of China; 3 School of Biomedical Engineering, Faculty of Medicine, Dalian University of Technology, No. 2, Linggong Road, Ganjingzi District, Dalian, 116024 Liaoning Province People’s Republic of China; 4 Department of Orthopedics, General Hospital of Northern Theater Command, No. 83, Wenhua Road, Shenhe District, Shenyang, 110000 Liaoning Province People’s Republic of China

**Keywords:** Spinal cord ischemia–reperfusion injury, Diallyl trisulfide, Mitochondrial function, Adenosine monophosphate activated protein kinase, Compound C

## Abstract

**Background:**

Spinal cord ischemia–reperfusion injury (SCII) is a catastrophic event, which can cause paraplegia in severe cases. In the reperfusion stage, oxidative stress was up-regulated, which aggravated the injury and apoptosis of neurons. As the main active ingredient of garlic, diallyl trisulfide (DATS) displays strong antioxidant capacity. However, it is unknown whether DATS can protect the neurons of SCII.

**Materials and methods:**

In this study, the descending aorta at the distal end of the left subclavian artery was ligated and perfused again after 14 min. Samples including blood and spinal cord (L2–L5) were taken 24 h later for morphological and biochemical examination.

**Results:**

After SCII, the rats showed motor dysfunction, increase apoptosis, malondialdehyde content, mitochondrial biogenesis and dynamic balance disorder. After the application of DATS, the adenosine monophosphate activated protein kinase (AMPK) was activated, the mitochondrial damage was improved, the oxidative stress was weakened, and the neuronal damage was recovered to some extent. However, the addition of compound C significantly weakened the protective effect of DATS.

**Conclusion:**

Oxidative stress caused by mitochondrial damage was one of the important mechanisms of neuronal damage in SCII. DATS could activate AMPK, stabilize mitochondrial biogenesis and dynamic balance, and reduce neuronal damage caused by oxidative stress.

## Introduction

In some thoracic and abdominal tumor surgery and spinal surgery, to reduce bleeding and facilitate surgery, it is usually necessary to block the aorta for a period of time before restoring perfusion. This procedure can result in spinal cord ischemia/reperfusion injury (SCII). It has been reported that 45% of SCII were due to iatrogenic causes, a catastrophic event that in severe cases could result in paraplegia, placing a significant financial and emotional burden on patients and their families [1]. SCII mainly consists of two stages of ischemia and reperfusion. Ischemia occurs during occlusion of major arteries or circulatory arrest, and reperfusion causes the release of reactive oxygen species and inflammatory cytokines and additional cell death [2]. The pathological mechanism of SCII is complex and not fully understood. Wang et al. proposed that in the process of reperfusion, a large amount of reactive oxygen species (ROS) was produced, the balance between ROS and antioxidants was lost, and oxidative stress was up-regulated, which promoted the damage and apoptosis of neurons [3]. Therefore, effective reduction of oxidative stress may be the key to neuroprotection in SCII.

Garlic is a common food in daily life, and its medical effects have long been documented, including anti-diabetes, kidney protection, anti-atherosclerosis, and antibacterial effects [4]. Besides, studies suggested that regular intake of garlic promoted antioxidant activity in the body [5]. Organic sulfide is the main active component of garlic, among which diallyl trisulfide (DATS) is one of the components with the largest number of sulfur atoms and the strongest antioxidant ability. Studies have confirmed that after oral administration of DATS in rats, the brain neuroinflammatory injury and oxidative stress induced by doxorubicin were attenuated indicating the neuroprotective effect of DATS [6]. In addition, the results showed that during the operation of rat skin flap, DATS reduced apoptosis and oxidative stress and imporved the survival rate of skin flaps [7]. Furthermore, both in vitro cultures and in diabetic mouse models, DATS has been reported to relieve oxidative stress and angiotensin II (Ang II)-induced vascular remodeling by inhibiting mitochondrial fission [8].

Adenosine monophosphate activated protein kinase (AMPK) is a highly conserved serine/threonine protein kinase, which can regulate cell homeostasis through a variety of pathways when activated. There is evidence that the redox state of oxidative stress-disequilibrium can activate AMPK in the upstream, regulate the dynamic balance and biosynthesis of mitochondria, reduce the production of reactive oxygen species in mitochondria, and achieve antioxidation [9]. Hao et al. [10] have demonstrated that DATS inhibited mitochondrial fission by activating AMPK, blocked ROS production and inhibited the apoptosis of human umbilical vein endothelial cell induced by high glucose.

Therefore, the present study was aimed to test the hypothesis that DATS may improve mitochondrial function, relieve oxidative stress and improve SCII in rats by activating AMPK.

## Materials and methods

### Animals and study groups

The study was approved by the Animal Care Committee (General Hospital of Northern Theatre Command) and conformed to the Guide for the Care and Use of Laboratory Animals (NIH Publication No. 85-23, revised 1996).

Forty-eight male Sprague–Dawley (SD) rats aged 8 weeks and weighing 220–250 g were purchased from the HUAFUKANG Bioscience Co., Ltd, Beijing, China. They were maintained at a constant temperature (25 ± 1 °C) and humidity (50–60%) and raised in a light/dark cycle of 12/12-h with free access to standard animal chow and tap water. After 1-week acclimatization period, the rats were randomly assigned to 4 groups (n = 12 for each group): the sham group, the SCII group, the SCII + DATS group, and the SCII + DATS + compound C (CC) group. DATS (dissolved in corn oil, 40 mg/kg/day, C3079, APExBIO, Houston, TX, USA) was orally administered for 3 days before SCII and the last dose was given 10 min before reperfusion [11]. CC (0.25 mg/kg, HY-13418A, MedChemExpress, Monmouth Junction, NJ, USA) was injected via tail 10 min prior to reperfusion [12].

### Surgical procedures

The rats were placed on the heated operating table after anesthetizing with chloral hydrate (350 mg/kg, i.p.) and mechanically ventilated. The rectal temperature was measured and maintained at 37 ± 0.5 °C. To monitor proximal and distal blood pressure, catheters were inserted into the left carotid artery and the right femoral artery, respectively. An incision was made at the fourth intercostal level of the left chest to expose the descending aorta just distal to the left subclavian artery. To induce SCI, a surgical clip was used to clamp the aorta after systemic heparinization (200 IU/kg) and the femoral blood pressure maintained at 10 mmHg was confirmed as ischemia. After a 14-min ischemia, the clip was removed followed by reperfusion [13, 14]. The rats in the sham group went through the same process without clamping. During the experimental period, manual bladder expression was performed twice per day. 24 h after reperfusion, the rats, except those used for behavioral testing (n = 6 for each group), were euthanized for the blood and spinal cord (L2–L5) samples.

### Neurological function assessment

At the 1st, 3rd, 5th, and 7th days after ischemia–reperfusion, motor function of rats was assessed by the Basso, Beattie, and Bresnahan (BBB) locomotor scoring scale in a blinded manner. The rats were placed separately in an open large basin. The observer gently tapped the basin wall to make the rats crawl, and scored them according to their walking and coordination. The assessment included limb movement, extent of abdomen drag, trunk position and stability, claw position and placement, tail position, and gait, with a score of 0 indicating complete loss of motor function and a score of 21 indicating normal motor ability [15, 16].

### Serological experiment

The serum samples were separated by centrifugation at 4 °C, 1000 g for 10 min in anticoagulant tubes. The activity of superoxide dismutase (SOD) and glutathione peroxidase (GSH-PX), and the contents of malondialdehyde (MDA) and glutathione (GSH) were detected by colorimetric reagent kits. SOD decreased the nitro-blue tetrazolium from xanthine-xanthine oxidase system (A001-3, Jiancheng, Nanjing, China), MDA condensed with thiobarbituric acid (TBA) to form a red product (A003-1, Jiancheng, Nanjing, China), GSH reacted with 5-5-dithiobis 2-nitrobenzoic acid (DTNB) to form a yellow compound (A006-2-1, Jiancheng, Nanjing, China), and the above products have a peak absorption at 560, 532 and 405 nm respectively. Besides, GSH-PX assay kit (A005-1, Jiancheng, Nanjing, China) measured GSH-PX activity based on the consumption of GSH [17].

### Transmission electron microscopy (TEM)

The spinal cord tissues were carefully divided into small pieces (1 mm^3^) as previously described [18], and fixed by 2.5% glutaraldehyde at 4 °C overnight. Thereafter, the specimens were fixed with 1% osmium tetroxide for 1 h, and dehydrated with rising concentrations of ethanol solutions. Subsequently, they were embedded in epoxy resin, which were cut in ultrathin sections (60 nm) on a microtome (EM UC7, Leica, Vienna, Austria) and transferred to copper grids. Finally, the blocks were stained with 3% uranyl acetate and lead citrate. Images were captured under TEM (HI7700, Hitachi, Tokyo, Japan) with an operative voltage of 80 kV and analyzed using Image J software.

### Hematoxylin–eosin staining

Fresh spinal cord tissues were immersed in 4% formaldehyde for 24 h at room temperature and embedded in paraffin. Subsequently, they were sectioned at 5 μm thickness and being deparaffinized and rehydrated before histological analysis. The kit for hematoxylin–eosin (HE) staining was purchased from KeyGEN (KGA224, KeyGEN Bio TECH Corp., Jiangsu, China). As indicated in the instructions, the slices were stained with hematoxylin for 5 min, wash gently, and stained with eosin for 2 min. Then, the slices were dehydrated by graded ethanol, permeated in xylene and mounted with resin. Finally, the sections were observed and photographed by a UB203i microscope (Aopu Technology, Chongqing, China).

### Nissl staining

Prior to staining, sections were dewaxed and hydrated as in HE staining. Then, sections were stained with Nissl staining solution (G1036, Servivebio, Wuhan, China) for 10 min at room temperature and differentiated with 0.1% glacial acetic acid. The pictures could be taken when Nissl bodies in the cytoplasm of neuros were dark blue and the background was light blue.

### TUNEL staining

A TUNEL kit (abs50033, Absin, Shanghai, China) was used for the TUNEL assay. As mentioned above, spinal cord sections were deparaffinized and rehydrated. Following washing with PBS, the sections were treated with proteinase K (20 μg/mL) at room temperature for 20 min and washed again in PBS. Then the sections were protected from light and incubated in a TUNEL reaction buffer and TdT enzyme mix at for 2 h at 37 °C. After washing with PBS, sections were incubated with DAPI for 5 min. Finally, anti-fluorescence quenching agent was added and slices were observed using a confocal microscope (Nikon, Tokyo, Japan).

### Dihydroethidium staining

ROS contents were determined by the fluorescence probe dihydroethidium (DHE) (KGAF019, KeyGEN Bio TECH Corp., Jiangsu, China). According to the instructions, the frozen sections (8 μm thickness) were incubated with 20 μM operating fluid at 37 °C in the dark for 30 min. The fluorescence images were performed by a Nikon C2 Plus confocal microscope, and the fluorescence intensity, which represented ROS levels, was analyzed by using Image J software.

### Western blotting

Proteins were isolated from spinal cord using RIPA (abs9225, Absin, Shanghai, China) with protease inhibitor (BP101, Bio-Platform, Shanghai, China), and the concentration was determined with BCA Protein Assay Reagent (BP104, Bio-Platform, Shanghai, China). Protein samples were mixed with sample buffer and boiled for 10 min. For western blotting, samples containing 20 ug protein were loaded, separated by electrophoresis, and then transferred onto polyvinylidene difluoride membranes (0010, Merck Millipore Ltd., MA, USA) using sandwich method. An overnight incubation at 4 °C with primary antibodies against AMPK (5831, Cell Signaling Technology, Boston, MA, USA), p-AMPK (2535, Cell Signaling Technology, Boston, MA, USA), Drp1 (8570, Cell Signaling Technology, Boston, MA, USA), p-Drp1 (3455, Cell Signaling Technology, Boston, MA, USA) was performed before incubating with horseradish peroxidase-conjugated secondary antibodies at room temperature for 1 h. The protein bands were visualized by enhanced chemiluminescence (P10300, NCM, Suzhou, China) and photographed by an image system (5200, Tanon, Shanghai, China).

### Statistical analysis

The results were recorded as the mean ± SEM (standard error of the mean). All statistical analyses were performed using GraphPad software (v9.0, GraphPad Software, San Diego, CA, USA) based on the Student’s *t* test or one-way analysis of variance, followed by Bonferroni post hoc test. *P* < 0.05 was considered to indicate a statistically significant difference.

## Results

### DATS improved neurologic motor function of SCII rats

**Fig. 1:**
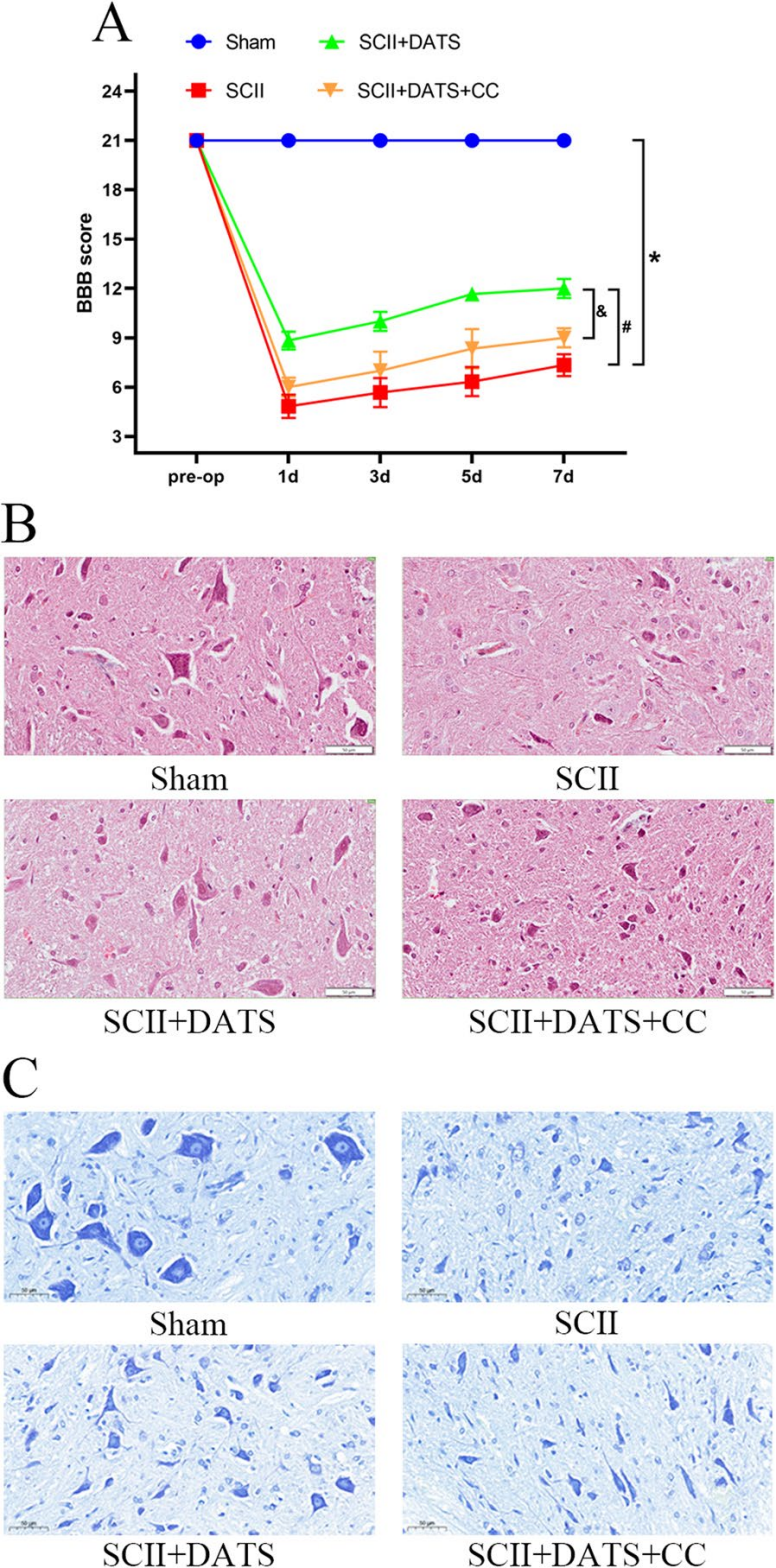
DATS improved neurologic motor function of SCII rats. **A** The Basso, Beattie, and Bresnahan (BBB) score of different groups. **B** Representative images of HE staining of spinal cord tissues. Bar = 50 μm. **C** Representative images of Nissl staining of spinal cord tissues. Bar = 50 μm. The data represent the mean ± SEM. *P < 0.05 vs. sham group. #*P* < 0.05 vs. SCII group. &*P* < 0.05 vs. SCII + DATS group. (n = 6)

The study used the BBB function scale to evaluate the motor function of rats. Compared with the Sham group, all rats undergoing SCII surgery showed significant neurological decline (Sham vs. SCII [*P* < 0.0001], Sham vs. SCII + DATS [*P* < 0.0001], Sham vs. SCII + DATS + CC [*P* < 0.0001]). However, DATS-treated rats showed less deterioration than the SCII group, and the decline was aggravated after DATS + CC treatment. 24 h later, the neurological function gradually recovered (Fig. 1A). The degree of neurological function recovery in the SCII + DAT group was significantly higher than that in the SCII group (*P* < 0.0001), while the degree of recovery in the DATS + CC group was decreased than the SCII + DATS group (*P* < 0.0001).

24 h after reperfusion, the spinal cord tissue was extracted for HE and Nissl staining. In the Sham group, the spinal cord motor neurons were characterized by clear outline, normal morphology, homogenous cytoplasm, and uniform distribution of granular Nissl’s bodies. In the SCII group, there was an obvious loss of motor neurons and a significant decrease in the number of Nissl’s bodies. The above pathological injuries were greatly alleviated after DATS treatment, and more intact motor neurons and more dark stained Nissl’s bodies could be observed. However, administration CC attenuated the therapeutic effect of DATS in the DATS + CC group (Fig. 1B, C).

### DATS ameliorated the apoptosis of spinal cord cells in SCII rats

**Fig. 2:**
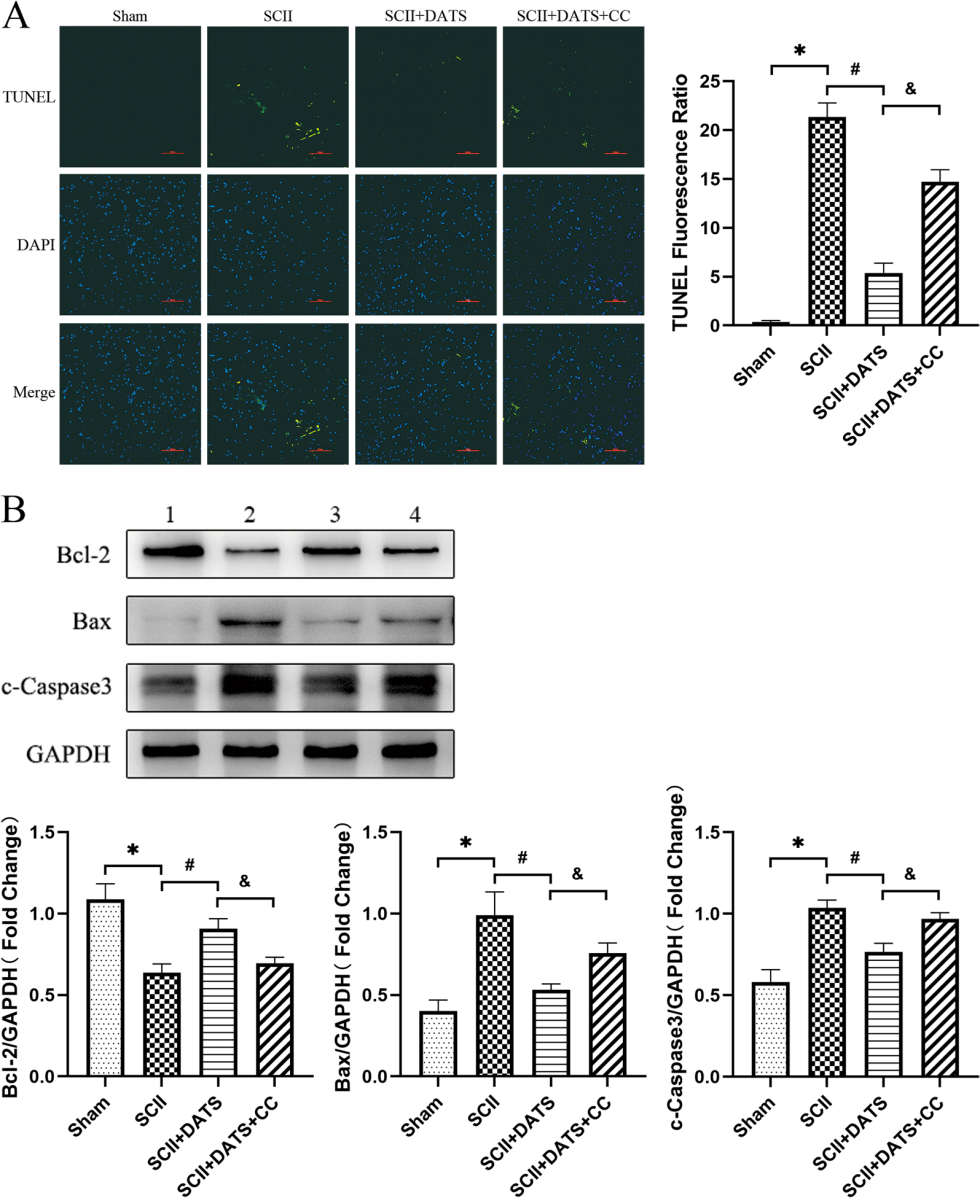
DATS ameliorated the apoptosis of spinal cord cells in SCII rats. **A** Representative images showing TUNEL staining and quantitative analysis of spinal cord tissues. Bar = 100 μm. **B** Representative western blot image and relative expression of Bcl-2, Bax, and c-Caspase3. Lane 1: sham group. Lane 2: SCII group. Lane 3: SCII + DATS group. Lane 4: SCII + DATS + CC group. The data represent the mean ± SEM. *P < 0.05 vs. sham group. #*P* < 0.05 vs. SCII group. &*P* < 0.05 vs. SCII + DATS group. (n = 6)

Ischemia–reperfusion injury leads to neuronal apoptosis [19], and we used the TUNEL protocol to evaluate the apoptosis of neurons (Fig. 2A) and Western blot to demonstrate the level of apoptosis-related proteins [20] (Fig. 2B). A marked increase of TUNEL-positive signal was detected after SCII (*P* < 0.0001), while signal was significantly reduced in rats of SCII + DATS (*P* < 0.0001). Similarly, the expression level of anti-apoptosis protein Bcl-2 was downregulated and the expression level of pro-apoptotic protein Bax and cleaved Caspase3 (c-Caspase3) were upregulated in SCII group (Bcl-2: *P* = 0.0150; Bax: *P* = 0.0213; c-Caspase3: *P* = 0.0070), while markedly reversed by DATS (Bcl-2: *P* = 0.0304; Bax: *P* = 0.0.74; c-Caspase3: *P* = 0.0186). Collectively, the results suggested a protective function of DATS against the apoptosis in response to SCII. Besides, the study showed the treatment of CC decreased the protective effect of DATS (TUNEL fluorescence ratio: *P* < 0.0001; Bcl-2: *P* = 0.0427; Bax: *P* = 0.0359; c-Caspase3: *P* = 0.0371).

### DATS alleviated oxidative stress in SCII rats

**Fig. 3:**
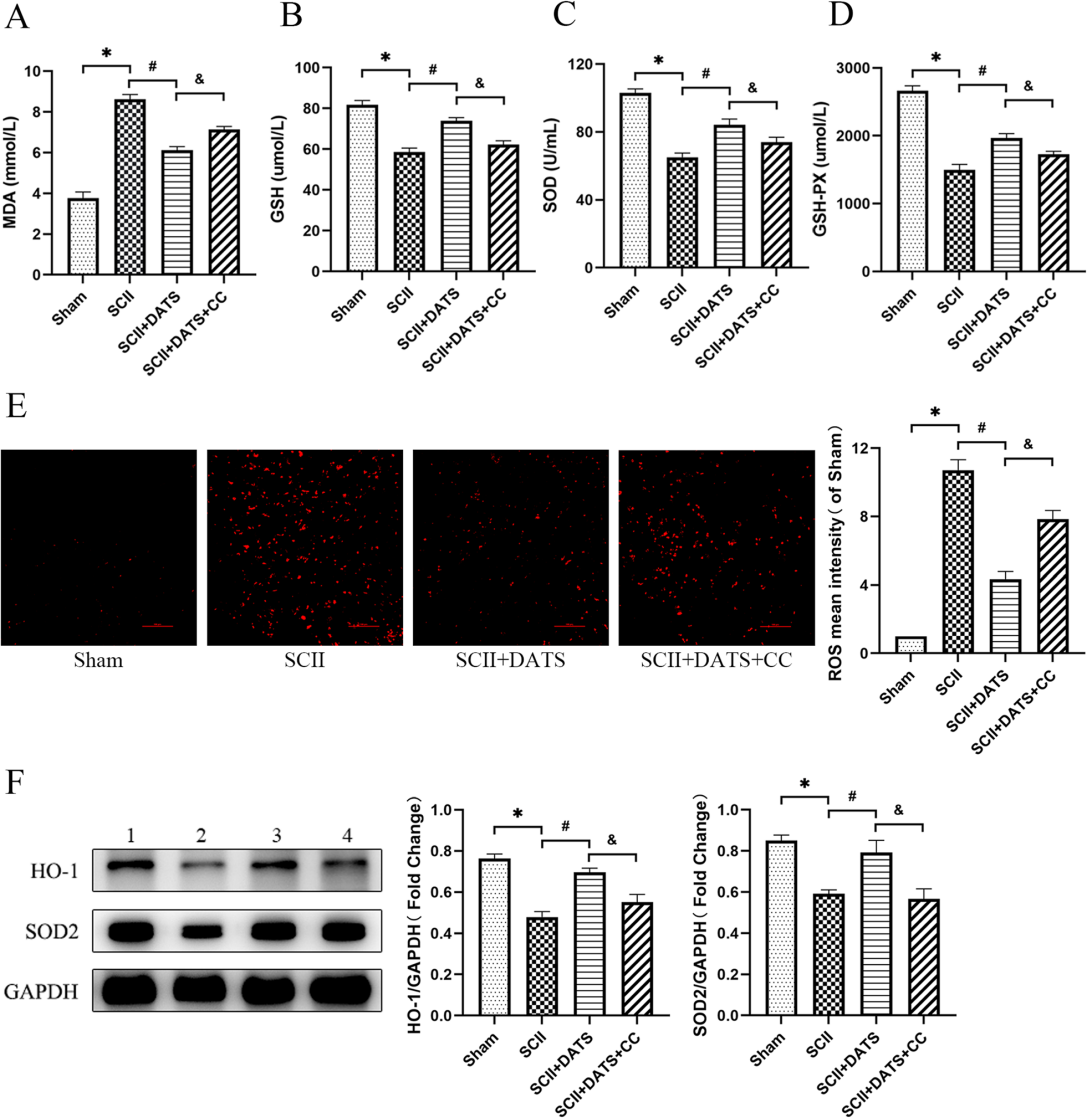
DATS alleviated oxidative stress in SCII rats. **A** The content of malondialdehyde (MDA). **B** The content of glutathione (GSH). **C** The activity of superoxide dismutase (SOD). **D** The activity of glutathione peroxidase (GSH-PX). **E** Representative images of reactive oxygen species (ROS) (red) by dihydroethidium staining and quantitative analysis of ROS mean intensity. Bar = 100 μm. **F** Representative western blot image and relative expression of HO-1 and SOD2. Lane 1: sham group. Lane 2: SCII group. Lane 3: SCII + DATS group. Lane 4: SCII + DATS + CC group. The data represent the mean ± SEM. *P < 0.05 vs. sham group. #*P* < 0.05 vs. SCII group. &*P* < 0.05 vs. SCII + DATS group. (n = 6)

MDA is one of the major lipid peroxides that reflects the level of oxidative stress and the degree of cell damage. GSH is an important non-enzymatic antioxidant in the body, and its content is an important factor to measure the antioxidant capacity. SOD and GSH-PX are the main scavenging enzymes of ROS in the cells, which reflecting the anti-oxidative stress ability. In SCII group, it indicated that the level of MDA was elevated (*P* < 0.0001), while the level of GSH (*P* < 0.0001) and the activities of SOD (*P* < 0.0001) and GSH-PX (*P* < 0.0001) were reduced. After treatment of DATS, it can be seen that the oxidative stress level decreased and the antioxidant stress ability increased (MDA: *P* < 0.0001; GSH: *P* < 0.0001; SOD: *P* = 0.0004; GSH-PX: *P* = 0.0004). However, in SCII + DATS + CC group, MDA content was higher than that in DATS group (*P* = 0.0004), while GSH content (*P* = 0.0002) and SOD (*P* = 0.0365) and GSH-PX (*P* = 0.0079) activities were lower than that in DATS group (Fig. 3A–D). Later, oxidative stress in spinal cord was detected (Fig. 3E). Fluorescence staining showed that the mean ROS intensity in SCII group was significantly rose (*P* < 0.0001), which could be reversed after DATS treatment (*P* < 0.0001), while the content of ROS increased again after DATS + CC treatment (*P* < 0.0001). Meanwhile, western blot analysis (Fig. 3F) suggested that the variation trend of heme oxygenase-1 (HO-1) and SOD2 expression levels in the rats was the same as that of SOD content in serological examination (HO-1: Sham vs. SCII [*P* = 0.0012], SCII vs. SCII + DATS [*P* = 0.0028], SCII + DATS vs. SCII + DATS + CC [*P* = 0.0266]; SOD2: Sham vs. SCII [*P* = 0.0014], SCII vs. SCII + DATS [*P* = 0.0321], SCII + DATS vs. SCII + DATS + CC [*P* = 0.0424]).

### DATS attenuated mitochondrial damage in SCII rats by activating AMPK

**Fig. 4:**
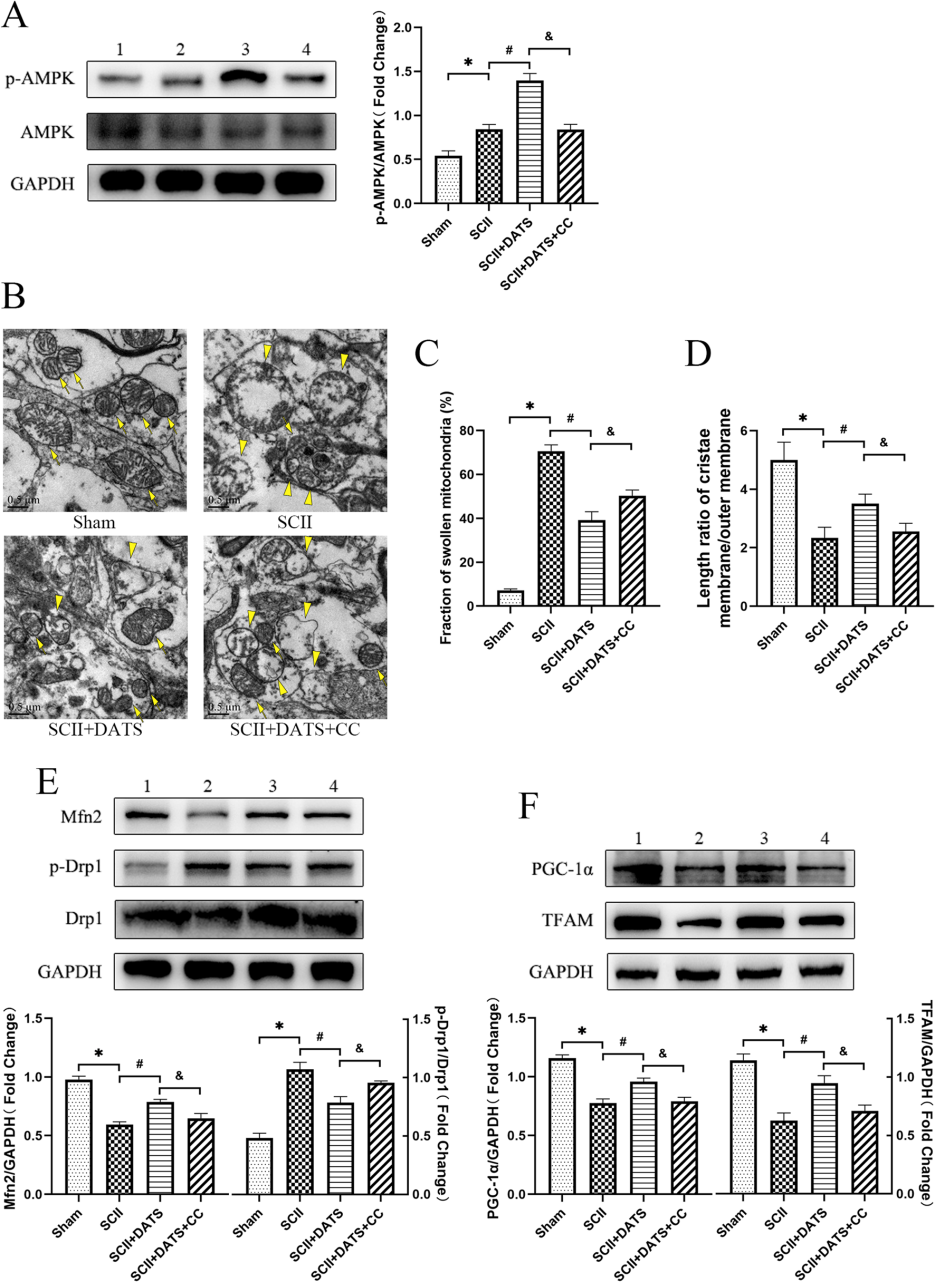
DATS attenuated mitochondrial damage in SCII rats by activating AMPK. **A** Representative western blot image and relative expression of p-AMPK. **B** Representative transmission electron microscopic images of spinal cord tissue. Bar = 0.5 μm. Arrows indicate the normal mitochondria, and arrowheads indicate the swollen mitochondria. **C** Fraction of swollen mitochondria. **D** Length ratio of the mitochondrial cristae membrane and outer membrane. **E** Representative western blot image and relative expression of Mfn2 and p-Drp1. **F** Representative western blot image and relative expression of PGC-1α and TFAM. Lane 1: sham group. Lane 2: SCII group. Lane 3: SCII + DATS group. Lane 4: SCII + DATS + CC group. The data represent the mean ± SEM. *P < 0.05 vs. sham group. #*P* < 0.05 vs. SCII group. &*P* < 0.05 vs. SCII + DATS group. (n = 6)

Immunoblotting of spinal cord tissue confirmed that after DATS treatment (Fig. 4A), the expression of p-AMPK increased sharply in rats undergoing SCII surgery (*P* = 0.0045), while CC, as an inhibitor of AMPK, exerted its inhibitory effect and significantly reduced the expression of p-AMPK (*P* = 0.0049). Morphological changes in mitochondria in spinal cord were detected by TEM. As shown in Fig. 4B, the mitochondria of Sham group were clear and intact with dense cristae. Compared to the Sham group, swelling and even cavitation of mitochondria in SCII group was obvious, and cristae was fuzzy and disappeared. As a result (Fig. 4C, D), the fraction of swollen mitochondria rose (*P* < 0.0001) and the length ratio of cristae membrane to outer membrane decreased (*P* = 0.0018). Fortunately, the SCII + DATS group showed slighter mitochondria damage and occasionally blurred mitochondrial (the fraction of swollen mitochondria: *P* < 0.0001; the length ratio of cristae membrane to outer membrane: *P* = 0.0289). However, addition of CC significantly reduced this effect (the fraction of swollen mitochondria: *P* = 0.0284; the length ratio of cristae membrane to outer membrane: *P* = 0.0415).

Meanwhile, mitofusin 2 (Mfn2) and dynamin-related protein 1 (Drp1) were used to as markers of mitochondrial dynamic, and PPARγ coactivator-1-α (PGC-1α) and transcription factor A of mitochondrial (TFAM) were chosen to indicate biogenesis. The results of western blotting demonstrated that in SCII group (Fig. 4E, F), the expression of Mfn 2, PGC-1α and TFAM were decreased while the expression of phosphorylation of Drp1 (p-Drp1) was increased, showing the impairment of mitochondrial dynamic balance and biogenesis (Mfn 2: *P* = 0.0005; PGC-1α: *P* = 0.0012; TFAM: *P* = 0.0037; p-Drp1:* P* = 0.0014). Consistent with the previous results, treatment of DTAS ameliorated the change trend of mitochondria caused by ischemia–reperfusion injury (Mfn 2: *P* = 0.0035; PGC-1α: *P* = 0.0185; TFAM: *P* = 0.0239; p-Drp1:* P* = 0.0245), while the improvement in SCII + DATS + CC group was inhibited (Mfn 2: *P* = 0.0373; PGC-1α: *P* = 0.0228; TFAM: *P* = 0.0420; p-Drp1:* P* = 0.0378).

## Discussion

In the present study, we further demonstrated that the protective effects of DATS against SCII were associated with inhibiting oxidative stress. More importantly, CC blocked AMPK activation and inhibited the antioxidative effects of DATS.

SCII is a devastating event that causes a complex cascade of molecular and cellular events leading to sensory and motor impairments. The occurrence of SCII is associated with many pathophysiological conditions, such as thoracoabdominal aneurysm repair, intraspinal surgery, hypotension, degenerative cervical spondylotic myelopathy, etc. [21]. Despite great progress in perioperative strategies and surgical techniques, postoperative neurological deficits caused by SCII are still as high as 9–16% including many cases of paraplegia [1]. Therefore, the current research focus is to study the molecular mechanism of SCII and find effective neuroprotective measures. There are two main mechanisms involved in SCII. When the spinal cord is deprived of blood supply, the oxygen supply is reduced and irreversible primary damage occurs. Subsequently, with the occurrence of reperfusion, ROS accumulate and oxidative stress intensifies, leading to additional cell death [22], so oxidative stress plays an important role in SCII. This was confirmed by both serological examination and tissue staining in our experiment. Compared to the sham group, the oxidative stress factor MDA was significantly increased in SCII group rats, while the antioxidant GSH, SOD and GSH-PX were decreased, and the ROS intensity was rose.

As one of the main sources of ROS, mitochondrial structure and dysfunction are considered to be one of the key pathological changes in SCII [23]. In order to meet different cellular energy and metabolic requirements, cells regulate mitochondrial biogenesis through PGC-1α and TFAM. Moreover, mitochondria are dynamic organelles, which are in a dynamic balance of constant fission and fusion. Governed by related proteins containing Mfn2 and Drp1, mitochondria maintain a relatively stable size and morphology [24]. Our study found that after SCII surgery, mitochondrial morphology changed with obvious swelling and disappearance of cristae. At the same time, the expression of TFAM and PGC-1α decreased, indicating that biogenesis became chaotic, and Drp1 and Mfn2 were expressed in opposite directions after SCII, indicating that mitochondrial dynamic balance was disturbed.

As the main active ingredient of garlic, DATS has attracted the attention of many researchers. Its antibacterial, anticancer, immunomodulatory and cardioprotective effects have received a large number of studies especially in the aspect of anti-oxidative stress [25]. In mice with concanavalin A-induced acute liver injury, DATS provided effective protection against hepatocytes by inhibiting oxidative stress [26]. In the mouse model of liver and lung injury induced by naphthalene, the application of DATS effectively reduced the level of MDA in tissues, increased the level of SOD and GSH, and relieved the inflammation of liver and lung [27]. In addition, the study found that DATS regulated the intracellular redox status by activating AMPK, and blocked the cell cycle, thus inhibiting the growth of human gastric adenocarcinoma cells [28]. Also, another study confirmed that AMPK activation was very important to inhibit the production of excess mitochondrial ROS during reperfusion in a mouse model of myocardial ischemia–reperfusion injury. AMPK agonist AICAR inhibited mitochondrial fusion and had a beneficial effect on its dynamics, ultimately reducing myocardial infarction size and improving left ventricular function [29]. Based on this, we assumed that DATS could reduce mitochondrial damage caused by SCII and reduce ROS levels by activating AMPK, thus having a protective effect on neurons. Fortunately, our results were consistent with the assumption that under the treatment of DATS, the expression of AMPK increased significantly, the mitochondrial damage caused by SCII was alleviated, the level of ROS decreased, and the morphology and function of neurons improved. Meanwhile, to better validate this hypothesis, AMPK inhibitor CC was added. The results showed that after simultaneous application of DATS and CC, the activation of AMPK was suppressed, and the protective effect of DATS on SCII was significantly weakened.

Our findings suggested that DATS might serve as a potent protective agent against SCII. However, several limitations of the study exist. The sample size number of this study was small and the follow-up period was short. Our further research will expand the animal number and prolong the observation period to better corroborate the hypothesis. Moreover, more in vitro experiments are needed to better understand the molecular mechanisms related to SCII and to better verify the protective effect of DATS.

## Conclusion

In conclusion, our study demonstrated that oxidative stress caused by mitochondrial damage was one of the important mechanisms of neuronal damage in SCII, and DATS, as an antioxidant, could activate AMPK, stabilize mitochondrial biogenesis and dynamic balance, and reduce neuronal damage caused by oxidative stress. DATS may be identified as a therapeutic approach against SCII, but further studies are necessary in more depth.

## Data Availability

The datasets used and/or analyzed during the current study are available from the corresponding author on reasonable request.
